# HBV Serologic Testing Practices and Reactivation Outcomes Before Rituximab Initiation: A Retrospective Observational Study at an Academic Tertiary Care Centre

**DOI:** 10.3390/jcm15155762

**Published:** 2026-07-23

**Authors:** Ahmed S. Barefah, Omar M. Raslan, Hatem M. Alahwal, Eman M. Mansory, Osman O. Radhwi, Esraa Almutairi, Lujain Alsayegh, Lujain Alrabghi, Basmah S. Almutairi, Mohammed A. Alsaadi, Salem M. Bahashwan

**Affiliations:** 1 Hematology Department, Faculty of Medicine, King Abdulaziz University Hospital, King Abdulaziz University, Jeddah 21589, Saudi Arabiaemmansory@kau.edu.sa (E.M.M.);; 2 Hematology Research Unit, King Fahd Medical Research Center, King Abdulaziz University, Jeddah 21585, Saudi Arabia; 3 Department of Internal Medicine, University of Jeddah, Jeddah 21493, Saudi Arabia; omraslan@uj.edu.sa; 4 Department of Internal Medicine, Faculty of Medicine, King Abdulaziz University Hospital, King Abdulaziz University, Jeddah 21589, Saudi Arabia; esraaalmutairi@gmail.com (E.A.); basma-salman@hotmail.com (B.S.A.)

**Keywords:** hepatitis B virus, HBV reactivation, rituximab, lymphoma, antiviral prophylaxis, screening, Saudi Arabia

## Abstract

**Background/Objectives**: Hepatitis B virus (HBV) infection remains a major global health challenge because of its association with cirrhosis, hepatic failure, and hepatocellular carcinoma. Patients receiving rituximab-containing regimens are at particularly high risk for HBV reactivation due to profound B-cell depletion and prolonged immunosuppression. This study aimed to evaluate HBV serologic testing practices before rituximab initiation and to assess the incidence and outcomes of HBV reactivation among patients treated with rituximab at a tertiary care center in Jeddah, Saudi Arabia. **Methods**: A retrospective observational study was conducted on patients who received rituximab between 2010 and 2020 at a tertiary university hospital in Jeddah, Saudi Arabia. Demographic, clinical, laboratory, and treatment-related data were collected from electronic medical records. HBV reactivation was defined according to the American Association for the Study of Liver Diseases (AASLD) criteria. **Results**: A total of 611 patients were included. HBV serologic testing prior to rituximab initiation was documented in 60.8% of patients with hematological malignancies and 71.7% of those with non-hematological diseases. HBV reactivation occurred in 7 patients (3.4%) in the hematological malignancy cohort, all with lymphoma. Nine patients received antiviral prophylaxis. HBV reactivation was associated with significant morbidity, and liver-related mortality occurred in 28% of reactivation cases. **Conclusions**: HBV serologic testing was not universally documented prior to rituximab initiation, and antiviral prophylaxis was received by few patients. HBV reactivation was associated with significant morbidity and mortality. These findings support the need for systematic HBV evaluation and prophylaxis protocols in patients receiving rituximab.

## 1. Introduction

Hepatitis B virus (HBV) infection remains a major global public health challenge and is a well-recognized cause of chronic liver disease, cirrhosis, and hepatocellular carcinoma [1,2]. Chronic HBV infection is defined by the persistence of hepatitis B surface antigen (HBsAg) in serum for at least six months [3]. According to the World Health Organization (WHO), approximately 254 million individuals were living with chronic HBV infection in 2022, with an estimated 1.1 million HBV-related deaths annually, mainly due to cirrhosis and hepatocellular carcinoma [4].

In Saudi Arabia, the prevalence of HBV infection has significantly declined over the past decades following the implementation of universal vaccination programs. Nevertheless, HBV infection continues to represent an important healthcare burden, with an estimated prevalence of approximately 1.7% in recent epidemiological studies [5].

HBV reactivation is a potentially life-threatening complication that may occur in patients receiving immunosuppressive therapies, particularly rituximab-containing regimens [6,7]. Reactivation may occur in patients with chronic HBV infection as well as those with resolved HBV infection. Clinically, HBV reactivation may range from asymptomatic elevation of liver enzymes to fulminant hepatic failure and death [8].

Rituximab is a chimeric monoclonal antibody directed against the CD20 antigen expressed on B lymphocytes and has become a cornerstone therapy for multiple hematological malignancies and autoimmune diseases [9,10,11,12]. However, rituximab induces prolonged B cell depletion and impaired humoral immunity, substantially increasing the risk of HBV reactivation [13].

Several studies have demonstrated that antiviral prophylaxis significantly reduces HBV reactivation, hepatitis-related complications, and treatment interruptions in patients receiving rituximab-containing therapy [14,15,16,17]. Consequently, major international societies including the American Association for the Study of Liver Diseases (AASLD), European Association for the Study of the Liver (EASL), American Society of Clinical Oncology (ASCO), and Asian Pacific Association for the Study of the Liver (APASL) recommend universal HBV screening prior to initiation of rituximab therapy [8,18,19,20].

Despite these recommendations, adherence to HBV screening and prophylaxis protocols remains inconsistent in routine clinical practice. Data regarding HBV screening and reactivation in Saudi Arabia remain limited.

The primary objective of this study was to evaluate HBV serologic testing practices prior to rituximab initiation at a tertiary care center in Jeddah, Saudi Arabia. Secondary objectives included assessment of HBV reactivation incidence, associated morbidity and mortality, and identification of potential risk factors associated with HBV reactivation.

## 2. Methods

### 2.1. Study Design and Setting

This retrospective observational study was conducted at a tertiary university hospital in Jeddah, Saudi Arabia. Patients who received rituximab between January 2010 and December 2020 were identified through the institutional electronic medical record system. The study was approved by the institutional Unit of Biomedical Ethics (Reference No.: 711-19). Due to the retrospective nature of the study, informed consent was waived.

### 2.2. Study Population

We included all patients aged 1 year or older who received at least one dose of rituximab during the study period, regardless of indication. Patients with previously diagnosed chronic liver disease prior to rituximab initiation were excluded.

### 2.3. Data Collection

Clinical and laboratory data were obtained from the hospital electronic medical record system. Collected variables included: demographic characteristics, nationality, indication for rituximab therapy, number of rituximab doses administered, concurrent chemotherapy or immunosuppressive therapy, HBV screening status, HBV serologic testing results, HBV DNA levels, alanine aminotransferase (ALT) levels, antiviral prophylaxis use, HBV reactivation outcomes and morbidity and mortality data. HBV screening included testing for HBsAg, anti-HBc, and anti-HBs using commercially available enzyme immunoassays. HBV DNA quantification was performed using real-time polymerase chain reaction (PCR) methodology (Bio-Rad Laboratories, Inc., Hercules, CA, USA). For the purpose of this study, “HBV screening performed” was defined as documentation of at least one HBV serologic marker (most commonly HBsAg) in the medical record prior to rituximab initiation; the number of patients tested for each individual marker (HBsAg, anti-HBc, anti-HBs) separately was not consistently available for the full cohort and could not be reliably reconstructed, which is acknowledged in the Section 5.

### 2.4. Definition of HBV Reactivation

HBV reactivation was defined according to AASLD guidelines. In patients with resolved HBV infection (HBsAg-negative and anti-HBc-positive), reactivation was defined as either detection of HBV DNA in serum, or reverse seroconversion with reappearance of HBsAg [18].

### 2.5. Study Outcomes

Primary outcomes were the documentation rate of HBV serologic testing prior to rituximab initiation and incidence of HBV reactivation among patients receiving rituximab. Secondary outcomes were morbidity associated with HBV reactivation and mortality associated with HBV reactivation.

### 2.6. Statistical Analysis

Statistical analysis was performed using the SPSS version 27 (IBM Co., Armonk, NY, USA). Descriptive statistics were used to summarize demographic and clinical characteristics. Demographic variables were compared between patients with hematological malignancies and those with non-hematological diseases using the independent-samples *t*-test, as appropriate. Continuous variables were reported as means with ranges, while categorical variables were summarized as frequencies and percentages. Categorical variables were compared using the chi-square test; Fisher’s exact test was used instead where expected cell counts were small, including for comparisons involving HBV reactivation given the limited number of events. Univariate analysis was performed to evaluate factors associated with HBV reactivation. Odds ratios (ORs), 95% confidence intervals (CIs), and *p*-values were calculated. A *p*-value < 0.05 was considered statistically significant.

## 3. Results

### 3.1. Patient Characteristics

A total of 611 patients who received rituximab during the study period were included. Among the study population, 368 patients (60.2%) were female and 243 (39.8%) were male. Patient age ranged from 4 to 98 years. Saudi nationals accounted for 317 patients (51.9%), while 294 patients (48.1%) were non-Saudi. The number of rituximab doses administered ranged from 1 to 18 doses.

Among patients with hematological malignancies, the mean age was 54.8 years, compared with 40.0 years among patients with non-hematological diseases (*p* < 0.001).

Most patients with hematological malignancies received rituximab in combination with chemotherapy, whereas patients with non-hematological diseases more commonly received rituximab with immunomodulatory agents or corticosteroids. No patients had documented intravenous drug use. Table 1 shows the baseline characteristics of the study population.

### 3.2. HBV Screening and Reactivation

HBV screening prior to rituximab initiation was performed in only 124 patients (60.8%) with hematological malignancies and 292 patients (71.7%) with non-hematological diseases. Overall, 195 patients (31.9%) did not undergo HBV screening prior to rituximab therapy. Only 9 patients in the study cohort received prophylactic antiviral therapy. It should be noted that the number of patients who met formal criteria for antiviral prophylaxis based on baseline HBV serostatus could not be determined from the available data; therefore, it is not possible to calculate the proportion of eligible patients who received prophylaxis or to characterize prophylaxis practices as adherent or non-adherent to guideline recommendations.

HBV reactivation occurred in 7 patients (3.4%) among the hematological malignancy cohort. This denominator (7/204) reflects all patients with hematological malignancies who received rituximab, irrespective of screening status; among the subset who actually underwent HBV screening, the reactivation rate was higher, at 5.6% (7/124). All reactivation events occurred in patients with lymphoma.

Among patients who experienced HBV reactivation, 3 had received antiviral prophylaxis prior to or during rituximab therapy; entecavir and lamivudine were the antiviral agents used in the cohort overall. The available data do not allow determination of whether reactivation in these 3 patients occurred despite appropriate and timely prophylaxis, following delayed or abbreviated prophylaxis, in the context of poor adherence, or due to antiviral resistance. This finding should therefore not be interpreted as prophylaxis failure.

### 3.3. Morbidity and Mortality

Among the 7 patients with HBV reactivation, 2 patients (28%) developed liver-related complications and these 2 patients (28%) died due to HBV-related complications.

One patient died secondary to severe HBV flare, while another died due to liver failure.

### 3.4. Risk Factors Associated with HBV Reactivation

Univariate analysis demonstrated that a higher cumulative number of rituximab doses was associated with increased risk of HBV reactivation (OR 1.7, 95% CI 0.99–3.0, *p* = 0.05). No statistically significant association was observed between HBV reactivation and age, gender, nationality, or use of additional chemotherapy. Given the very small number of events (*n* = 7) and the inability to verify reference categories and event/non-event cell counts from the available dataset, the full univariate analysis has been removed. The risk factor findings described above should be regarded exclusively as exploratory observations.

## 4. Discussion

HBV reactivation remains a clinically significant and potentially life-threatening complication in patients receiving rituximab-containing regimens [6]. In this retrospective study from a tertiary care center in Jeddah, Saudi Arabia, we found that approximately one-third of patients had no documentation of any HBV serologic testing prior to rituximab initiation. Because the available data do not allow determination of which markers were tested in the remaining patients, a formal assessment of adherence to guideline-recommended screening (which requires at minimum HBsAg and anti-HBc) cannot be made; the findings reflect the rate of documented HBV serologic testing rather than verified guideline adherence.

Despite well-established international recommendations advocating universal HBV screening before anti-CD20 therapy, screening practices in real-world settings remain inconsistent. Our findings are concerning because rituximab is recognized as one of the highest-risk immunosuppressive agents for HBV reactivation. The incidence of HBV reactivation in our cohort of patients with hematological malignancies was 3.4%, and all reactivation events occurred among patients with lymphoma. This observation is consistent with previous reports demonstrating that lymphoma patients receiving rituximab-based chemotherapy are among the highest-risk populations for HBV reactivation [6,7].

Several mechanisms may explain the increased susceptibility to HBV reactivation in rituximab-treated patients. Rituximab induces prolonged B-cell depletion, resulting in impaired immune surveillance and reduced control of latent HBV infection. Furthermore, concomitant chemotherapy and corticosteroid exposure may further contribute to immunosuppression and viral replication [13].

Previous studies have demonstrated variable HBV reactivation rates depending on HBV serostatus, prophylaxis use, and duration of immunosuppressive therapy. In a randomized controlled trial, Huang et al. demonstrated that entecavir prophylaxis significantly reduced HBV reactivation among lymphoma patients receiving rituximab-containing chemotherapy [14]. Similarly, multiple meta-analyses have confirmed the protective effect of antiviral prophylaxis in high-risk patients [15,16,17].

Only a small number of patients (*n* = 9) received antiviral prophylaxis prior to rituximab therapy. However, because baseline HBV serostatus and the number of patients meeting formal prophylaxis criteria were not available from the retrospective dataset, it is not possible to calculate a prophylaxis adherence rate or to characterize these findings as suboptimal relative to guideline recommendations. The low absolute number of patients receiving prophylaxis may reflect under-recognition of reactivation risk, incomplete documentation, or other factors that cannot be distinguished from the available data. Prophylactic antiviral therapy was not statistically associated with reduced HBV reactivation risk in our analysis; however, this finding should be interpreted cautiously given the very small number of events and the inability to account for prophylaxis eligibility. Existing evidence from randomized trials and meta-analyses strongly supports the use of antiviral prophylaxis in patients at risk of HBV reactivation, and the absence of a statistically significant protective effect in this study should not be taken as evidence against prophylaxis.

It should be emphasized that only seven HBV reactivation events were observed in the entire cohort, and the univariate risk factor analysis must therefore be interpreted with considerable caution. Several odds ratios were accompanied by wide 95% confidence intervals (e.g., nationality, 95% CI 0.3–17.6; odds of death among patients with HBV reactivation, 95% CI 0.45–35.8), reflecting substantial statistical imprecision given the small number of events. The association between cumulative rituximab dose number and HBV reactivation was of borderline significance (OR 1.7, 95% CI 0.99–3.0, *p* = 0.05) and should be regarded as exploratory and hypothesis-generating rather than confirmatory. Similarly, the analysis of mortality risk associated with HBV reactivation is based on a very small number of deaths and is not statistically robust. These findings should not be interpreted as establishing causal or predictive relationships and require confirmation in larger, prospective cohorts.

Importantly, HBV reactivation in our study was associated with substantial clinical consequences, including liver-related complications and mortality. These findings underscore that HBV reactivation is not merely a biochemical event but may result in severe hepatic injury, interruption of chemotherapy, liver failure, and death.

Current international guidelines from the AASLD, EASL, ASCO, and APASL uniformly recommend HBV screening using HBsAg and anti-HBc prior to initiation of rituximab or other B-cell-depleting therapies [8,18,19,20].

Patients with chronic HBV infection or resolved HBV infection receiving high-risk immunosuppressive therapy should receive prophylactic antiviral therapy with high-potency agents such as entecavir or tenofovir [21,22,23,24,25].

Our findings have important implications for clinical practice in Saudi Arabia and the wider region. The relatively low screening and prophylaxis rates observed in this study highlight the need for standardized institutional HBV screening protocols, electronic medical record alerts, physician education initiatives and improved multidisciplinary collaboration between hematology, oncology, hepatology, and infectious disease services.

## 5. Limitations

This study has several limitations. First, the retrospective single-center design may limit generalizability. Second, incomplete HBV screening prior to rituximab initiation may have resulted in underestimation of occult HBV infection and HBV reactivation events. Third, the relatively small number of HBV reactivation cases limited the ability to perform robust multivariate analysis and lymphocyte counts and the duration or severity of lymphopenia were not systematically analyzed, which may have limited assessment of immune suppression as a potential contributor to HBV reactivation. Fourth, variability in follow-up duration and monitoring practices may have affected the detection of delayed HBV reactivation; the exact follow-up duration and the frequency of HBV DNA and liver function monitoring were not systematically recorded in the medical records and could not be reliably reconstructed for this retrospective analysis. Fifth, detailed baseline HBV serostatus (e.g., the number of patients who were HBsAg-positive, HBsAg-negative/anti-HBc-positive, or HBV DNA-positive at baseline) and the number of patients tested for each individual HBV marker were not consistently documented and are not reported separately; consequently, the number of patients who met formal criteria for antiviral prophylaxis, as opposed to the number who actually received it, could not be determined. Sixth, detailed case-level data for the seven patients with HBV reactivation (including baseline HBV DNA, ALT trends, exact timing of antiviral prophylaxis relative to rituximab initiation, and reasons for prophylaxis failure where applicable) were incompletely captured in the available records and are therefore not presented in case-level detail. Seventh, the cohort spanned a wide age range (4–98 years) and therefore included pediatric patients; the exact number of pediatric patients and a pediatric-specific sensitivity analysis are not reported, and findings should be interpreted with this age heterogeneity in mind. These gaps reflect limitations of the underlying retrospective dataset rather than omissions in reporting, and we have avoided supplementing them with data not present in the original records. Eighth, regarding data availability: the original patient-level database remains accessible to the study team; however, the specific data fields required to address several reviewer requests (HBV serology by individual marker, antiviral prophylaxis timing and eligibility, case-level reactivation details, and follow-up duration) were not prospectively captured in the data collection instrument used for this study, and therefore could not be extracted even from the original database. Future prospective studies with structured, comprehensive data collection incorporating these variables are needed to address these gaps.

## 6. Conclusions

HBV reactivation remains an important and preventable complication among patients receiving rituximab-containing therapy. Documentation of HBV serologic testing prior to rituximab was incomplete in approximately one-third of patients, and very few patients received antiviral prophylaxis; however, because complete screening marker data and prophylaxis eligibility criteria were not available, formal adherence to guideline recommendations could not be assessed. HBV reactivation was associated with significant morbidity and mortality, particularly among lymphoma patients receiving rituximab-containing regimens. Given the incomplete screening coverage and the small number of observed events, the reactivation rate reported here should be regarded as the detected rate within this retrospective cohort and may underestimate the true incidence; the risk-factor findings should likewise be viewed as hypothesis-generating rather than definitive.

Our findings support implementation of standardized institutional HBV screening and prophylaxis protocols before rituximab initiation. Greater adherence to international guideline recommendations may reduce preventable complications and improve patient outcomes. Prospective multicenter studies from Saudi Arabia and the Middle East are warranted to better define regional HBV reactivation risk and optimize preventive strategies.

## Figures and Tables

**Table 1:** Baseline characteristics of the study population.

		Hematological Malignancies		Non-Hematological Diseases		*p* Value
		Mean	Range	Mean	Range	
Age		54.8	5–98	40.0	4–92	<0.001
Number of Rituximab doses		6.7	1–18	4.3	1–10	<0.001
Number of cycles		6.2	1–18	2.2	1–6	<0.001
		Frequency	Percentage	Frequency	Percentage	
Gender	Male	123	(60.3%)	120	(29.5%)	<0.001
	Female	81	(39.7%)	287	(70.5%)	
Nationality	Saudi	64	(31.4%)	253	(62.2%)	<0.001
	Non-Saudi	140	(68.6%)	154	(37.8%)	
IV drug abuse	Yes	0	0	0	0	N/A
	No	204	(100.0%)	407	(100.0%)	
HBV screening performed	Yes	124	(60.8%)	292	(71.7%)	0.008
	No	80	(39.2%)	115	(28.3%)	
Other chemotherapy/immunomodulatory agents	Rituximab alone	0	(0.0%)	58	(14.3%)	
	Rituximab + high-dose steroid only	1	(0.5%)	62	(15.2%)	
	Rituximab + other chemo agents	202	(99.0%)	17	(4.2%)	
	Rituximab + immunomodulatory agents	0	(0.0%)	155	(38.1%)	
	Rituximab + immunomodulatory agent + high-dose steroid	1	(0.5%)	115	(28.3%)	

N/A: not applicable.

## Data Availability

The original contributions presented in this study are included in the article. Further inquiries can be directed to the corresponding author.
